# Immune check point inhibitors-induced hypophysitis: a retrospective analysis of the French Pharmacovigilance database

**DOI:** 10.1038/s41598-019-56026-5

**Published:** 2019-12-19

**Authors:** Julie Garon-Czmil, Nadine Petitpain, Franck Rouby, Marion Sassier, Samy Babai, Mélissa Yéléhé-Okouma, Georges Weryha, Marc Klein, Pierre Gillet

**Affiliations:** 1 Department of Clinical Pharmacology and Pharmacovigilance, University Hospital of Nancy Brabois, Biologie Médicale & Biopathologie, Rue du Morvan, 54511 Vandoeuvre Lès Nancy, France; 2 Department of Endocrinology and Medical Gynecology, University Hospital of Nancy, rue du Morvan, 54500 Vandœuvre-lès-Nancy, France; 3 UMR 7365 CNRS-Université de Lorraine, IMoPA, Campus Brabois Santé, 9 Avenue de la forêt de Haye, BP 20199, 54505 Vandœuvre-lès-Nancy, France; 4 Department of Clinical Pharmacology and Pharmacovigilance, University Hospital of Marseille, 270 Boulevard Sainte Marguerite, 13009 Marseille, France; 5 Department of Pharmacology, University Hospital of Caen, Avenue de la Côte de Nacre, 14000 Caen, France; 6 Department of Pharmacovigilance, Hôpital Henri Mondor, Assistance Publique Hôpitaux de Paris, Henri Mondor Hospital, 94010 Créteil, France

**Keywords:** Cancer immunotherapy, Multihormonal system disorders

## Abstract

Immune control point (ICI) inhibitors represent a significant advance in the management and survival of cancers such as melanoma or non-small cell bronchial carcinoma. However, they induce unusual side effects, such as hypophysitis, which are rarely described elsewhere. This nationwide retrospective study describes the characteristics of hypophysitis reported in the French pharmacovigilance database (FPVD). We requested for all cases of ICI-related hypophysitis registered in the FPVD before May 2018. An endocrinologist and a pharmacologist reviewed all cases. About 94 pituitary cases were selected, involving 49 females and 45 men. Ipilimumab alone or in combination was the most represented ICI (56%). Most cases (61%) were grade 3 severity and the majority (90%) were corticotropic deficiency cases. Cases with thyroid and/or gonadotropic involvement were 21% and 1% respectively. Five patients (8%) had panhypopituitarism. Pituitary MRI, when performed, was in favor of hypophysitis in 50%. No patient recovered his previous hormonal function. The mean time of onset was significantly shorter with ipilimumab than other ICIs. ICI-related hypophysitis generate deficits that do not spontaneously recover, even at a distance from the event, unlike thyroiditis. Patients must then benefit from long-term coordinated onco-endocrinological management, adapted to their own specific deficits.

## Introduction

Recently, immune checkpoint inhibitors (ICIs) have become the preferred treatment for many advanced solid tumours such as melanoma, non-small-cell lung cancer (NSCLC), and clear cell renal cancer^1^. Ipilimumab was the first authorized ICI, acting via cytotoxic T-lymphocyte antigen-4 (CTLA-4) blockade, followed by nivolumab and pembrolizumab, which both target the programmed death 1 receptor (PD-1), an effector ligand of the immune checkpoint pathway. More recently two other ICIs, atezolizumab and avelumab, which both target the PD1 ligand (PD-L1) have also been authorized for treating such diseases.

The mechanism of action of ICIs is to utilize the patient’s own immune system to destroy tumour cells or inhibit tumour growth, by restoring the decreased cellular immunity as well as boosting the production and proliferation of T lymphocytes. This ingenious mechanism of action has been shown to be efficient in terms of survival and disease-free survival but is jeopardized by the development of numerous and various immune related adverse effects (IRAEs), including endocrinological disorders as well as dermatological, digestive, cardiac, pulmonary, muscular, and haematological disorders, with all organs being potentially affected. Among the endocrinological damages, thyroiditis and hypophysitis seem to be the most common^2–7^. Unlike thyroiditis, hypophysitis does not commonly affect people and has dramatically emerged since the launch of ICI therapies^8–12^.

In order to achieve real-life data about the characteristics, diagnosis and management of this adverse effect related to the use of the most commonly used ICIs (ipilimumab, nivolumab and pembrolizumab), we performed a retrospective and descriptive analysis of cases of hypophysitis reported inadverse dru reaction the French network of regional pharmacovigilance centres since the marketing of these drugs.

## Materials and Methods

### Data collection

The data source used was the French Pharmacovigilance Database (FPVD)^13^. Briefly, the French Pharmacovigilance database was created in 1973 and, since 1985, all spontaneous reports of ADR have been recorded in the FPVD. According to French law, every health professional must report “serious” and/or “unexpected” adverse drug reactions to their regional centres of pharmacovigilance (31 in France). For each ADR report, information about the patient (age, sex, medical history), drug exposure (to the suspected drug and other associated non-suspected drugs) and ADR characteristics (“serious” or “non-serious”, “expected” or “unexpected”, causality score, outcomes) are recorded in the FPVD.

The FPVD was searched in April 2018 for all cases of endocrinological disorders (MedDRA system organ class (SOC) « endocrine disorders ») and hormonal biological abnormalities (within MedDRA SOC « investigations ») involving ipilimumab, nivolumab and/or pembrolizumab. No cases involving atezolizumab or avelumab were recorded. All anonymized recorded cases in the FPVD (General Data Protection Regulation Law) were reviewed by an endocrinologist and a pharmacologist to select confirmed hypophysitis cases and discard those with any aetiology other than iatrogenic. The French Pharmacovigilance Database was approved by the French Data Protection Agency (CNIL). No additional approval was required for studies based on this database, because all records respect the patient’s and notifier’s anonymity. We also attest that all our data originated from this database. Thus, this observational study did not require patient consent or ethics committee approval. Additionally, we certify that this study was approved by the French network of Regional Pharmacovigilance Centres.

### Case selection

Hypophysitis patients had to display (i) clinical symptoms such as cephalalgia associated with blurred vision, nausea or vomiting or clinical signs of anterior pituitary failure; and/or (ii) biological results consistent with a trophic hormone deficiency: prolactin (PRL), growth hormone (GH), adrenocorticotropic hormone (ACTH), thyroid-stimulating hormone (TSH), luteinizing hormone (LH) and/or follicle-stimulating hormone (FSH) in females, and testosterone in males; and/or (iii) abnormal MRI consistent with hypophysitis. Suspected cases of peripheral adrenal insufficiency without fludrocortisone supplementation were considered as adrenocorticotropic insufficiency secondary to hypophysitis^6^.

### Variables

We collected the characteristics of the patients (sex, age, previous immune disorder), suspected treatment (drug(s), dose, number of injections), hypophysitis (clinical symptoms, biological abnormalities, imaging, time to onset after start of ICI, outcomes), and patient’s medication (type of hormonal supplementation, weaning). The severity of hypophysitis was graded according to the CTCAE-NCI v4.0 (grade 1: asymptomatic patients, no treatment required; grade 2: symptoms not requiring hospitalization; grade 3: functional impairment limiting activities of daily living and requiring hospitalization; grade 4: life-threatening symptoms; and grade 5: death).

### Statistical analysis

The mean, standard deviation and median with minimal and maximal values were used to express quantitative variables, while qualitative variables were expressed with numbers and rates. The comparison of the averages was based on the ANOVA test together with Student’s t test when relevant. SAS software version 9·3 (SAS Institute, Cary, NY) was used to draw the analysis.

## Results

Among the 249 cases corresponding to an endocrinological disorder associated with ipilimumab, nivolumab or pembrolizumab, 94 corresponded to hypophysitis (Table 1). Among these latter cases, ipilimumab was the most-involved ICI, either alone (43%) or in combination with nivolumab (14%). Four patients received previous ICI treatment before the diagnosis (6·6%). The patients included 49 females and 45 males, whose mean age was 64·5 years (+/−14·1), and the most frequent indication was melanoma (74·5%). None of these patients displayed a previous immune disorder.

**Table 1:** Treatment characteristics, demographic and clinic-biological data of ICI-related hypophysites recorded in the French Pharmacovigilance Database (FPVD) until May 2018.

Patients	Immune Check Point Inhibitor(s) (ICI)			
	ipilimumab (ipi)	nivolumab (nivo)	Pembrolizumab (pembro)	ipilimumab + nivolumab (ipi + nivo)
Number of cases	40	28	13	13
Women n (%)	21 (52·5)	12 (49·9)	7 (53·8)	9 (69·2)
Age (mean ± SD) (years)	68·4 (±15·2)	62·8 (±9·8)	65·4 (±15·8)	55·5 (±13·7)
Body Mass Index (mean ± SD) (kg/m^2^)	27·1 (±4·9)	23·7 (±4·42)	22·3 (±3·9)	25·37 (±3·94)
Previous immunotherapy (n)	2	1	1	0
**ICI treatment (median [min-max])**				
Number of courses	4[1–7]	10[1–33]	7[1–19]	3[2–4]
Dose per course (mg)	235 [150–330]	242 [123–354]	136 [88–190]	ipi 74^60–90^ + nivo 229 [190–260]
Cumulated dose before hypophysitis (mg)	756 [495–1140]	1901 [294–8910]	616 [120–3610]	ipi 255 [146–300] + nivo 770 [438–924]
Time of onset (days)	79 [14–506]	155·5 [5–552]	184 [6–686]	119 [18–500]
**Indication**				
Melanoma	40	7	13	11
Non small cell type lung cancer	0	17	0	1
Small cell type lung cancer	0	1	0	0
Colorectal cancer	0	1	0	0
Renal cell carcinoma	0	2	0	0
Pleural mesothelioma	0	0	0	1
**Hypophysitis severity/outcome/MRI**				
Grade 1	0	0	0	0
Grade 2	9	4	4	4
Grade 3	17	22	9	9
Grade 4	5	2	0	0
Grade 5*	3	0	0	0
Recovered	1	0	1	0
MRI with pituitary enlargement	7	2	1	6
**Management**				
Levothyroxine supplementation	15	1	1	3
Hydrocortisone supplementation	32	28	13	12
Other supplementation	1	0	0	0
Immunotherapy continuation	4	14	8	9

Qualitative data are expressed as number and rates, and qualitative ones as mean (±SD) or median with [minimal and maximal values]. Gradation of severity is detailed in the Methods section. MRI: magnetic resonance imaging.

*Without enough evidence to exclude disease progression fatality.

Most of them (88%) expressed clinical symptoms, mainly fatigue and cephalalgia, justifying biological and morphological explorations and finally leading to the diagnosis of hypophysitis. Hyponatremia and arterial hypotension were exceptionally present. Five patients had pan-hypopituitarism. No patient had insipidus diabetes. Pituitary MRI results were available in 40 patients and only half of them demonstrated a hypophysitis aspect, whereas one patient had a pituitary micro-adenoma, another patient had an empty sella turcica, and the remaining patients had normal pituitary imaging. The only two patients with documented abnormal pituitary MRI recovered after hydrocortisone supplementation.

The median delay period for hypophysitis to develop, after the start of ICI treatment, was significantly shorter with ipilimumab alone or combined with nivolumab (83 days; min 14 - max 506) than with nivolumab or pembrolizumab alone (165 days; min 5 - max 686; p = 0·0001) (Table 1). The median number of cycles and the median cumulated dose before hypophysitis were not significantly higher for nivolumab than for the other drugs.

No cases corresponded to grade 1 hypophysitis. Three patients treated with ipilimumab with severe general state deterioration had rapid (24 to 48 hours) fatal outcome after hypophysitis diagnosis, which could eventually be considered as grade 5 hypophysitis, but without enough evidence to exclude disease progression fatality. One patient had concomitant myasthenia and died from respiratory failure. The second patient died from acute liver cytolysis and shock after decompensated adrenocorticotropic failure and the third died from melanoma progression. Nivolumab was also associated with severe (grade 4) hypophysitis in 2 patients of whom one finally died due to lung cancer progression. Patients treated with pembrolizumab had less severe disorders (grade 3 or 2), but one patient had fatal outcomes due to melanoma progression.

Hydrocortisone supplementation was provided to 85 patients (90·4%) and L-thyroxin supplementation to 20 patients (21.3%). Only one case of testosterone supplementation was started concomitantly with hydrocortisone for a man with panhypopituitarism, while in the other cases of panhypopituitarism, 3 women received hydrocortisone plus levothyroxine supplementation, and 1 man received hydrocortisone only. No patient was weaned from hormonal supplementation according to the last available information, except one male patient who had received short-term hydrocortisone treatment and thereafter displayed a normal ACTH stimulation test. The only patient who received no supplementation had her ICI treatment discontinued.

## Discussion

To the best of our knowledge, this series of 94 patients with ICI-induced hypophysitis is, as of today, the largest reported series. It provides complementary insights into the previously available data^3,4,11,14^ and case-series^8,10,15^, and, especially, reinforces clinical trial data^16^ by including patients with a potential previous ICI exposure or autoimmune history. In fact, no patient in our series had previous immune disease recorded in his or her medical file, which leads us to confirm, similarto the cas in other IRAEs, that the development of hypophysitis is not triggered by a pre-existing immune condition.

Hypophysitis was initially considered a specific IRAE of ipilimumab considering the presence of pituitary expression of CTLA-4 antigens^17^. Our series, with 41 cases (28 related to nivolumab and 13 to pembrolizumab), confirms previous literature reports showing that hypophysitis can also occur while anti PD-1^14^ and anti-PD-L1 are present^18^. It is, however, interesting to note that we observed a significantly shorter delay of onset with ipilimumab than with anti-PD1. Our results and literature data definitely illustrate that hypophysitis can occur regardless of the ICI target (CTLA4, PD1 or PD-L1) and its indication.

In accordance with literature stating that thyroid hormone deficiency appears to be less frequently reported than adrenocorticotropic or gonadotropic deficiencies, our series includes a larger proportion of patients requiring hydrocortisone supplementation than thyroidal supplementation. Clinical signs of adrenocorticotropic failure may be less often detected and underestimated for patients with advanced cancer with marked asthenia, nausea and vomiting. We observed that pituitary MRI contributed less to the findings of our cases than in the cases-series reported in the literature. Faje *et al*.^9^ reported that 100% of patients had abnormal MRI, but abnormalities were previously observed in 7/18 patients before symptoms occurred.

Hormonal supplementation weaning appears difficult to achieve, especially for hydrocortisone^19^ and this was confirmed in our series. However, we are also aware that weaning success assessments may be lowered by too short of a follow-up in prospective studies or a lack of information in retrospective studies; this phenomenon is one of the limitation of our study, with very few patients benefiting from a sufficient retrospective update.

Panhypopituitarism, although rare, should not be ignored. We observed that supplementation with gonadotropic hormones was rarely performed and we propose that it should be more often considered, especially in non-menopausal women to limit the consequences of oestrogens deficiency, such as osteoporosis. Considering the variable delay of hypophysitis development and the therapeutic regimens with ICI administrations every 2 or 3 weeks, the results of the clinical survey regarding hypophysitis appear to be helpful at any time of treatment, regardless of the ICI type. Delayed signs after treatment cessation can also occur^20^ and, therefore, hypophysitis should be kept in mind for any patient with present or past ICI exposure(s).

The main limitation of our work is the very probable underreporting of ICI-induced hypophysitis because these cases are now expected, especially non-severe cases (grades 1 and 2). This limitation persists despite the fact that since 2011, French health professionals have an obligation to report any suspected drug-induced adverse reaction, regardless of its expectedness or seriousness. Another main limitation is related to the retrospective collection of data, generating missing information, and thus potential uncertainties. However, the practitioners’ expertise in regional pharmacovigilance centres and the specific review of all cases by an endocrinologist warrant the accuracy of a diagnosis of hypophysitis and a good assessment of patients’ clinical histories and condition, but it must be acknowledged that, when detailed ACTH levels or stimulation tests to confirm primary *versus* secondary adrenal insufficiency were not available (10 cases), reliance on imperfect secondary markers was made. Therefore, it is possible that some misclassification occurred.

## Conclusion

Hypophysitis is definitely not a specific IRAE of ipilimumab treatment for melanoma. It occurs with any type of currently available ICI, anti-CTLA4, anti-PD1 or anti PD-L1, at any time of treatment and regardless of the type of cancer. Adrenocorticotropic deficiency may be more frequent than thyroidal deficiency and hormonal supplementation is required and has been proven to be difficult, if not impossible, to wean. Gonadotropic hormonal supplementation should probably be more often considered. The management of patients clearly requires long-term onco-endocrinological care.

## Data Availability

Restrictions apply to the availability of data generated or analyzed during this study because they were used under the authority of the French Drug Agency (ANSM). The corresponding author will on request detail the restrictions and any conditions under which access to some data may be provided.
